# Methods to evaluate the twin formation energy: comparative studies of the atomic simulations and in-situ TEM tensile tests

**DOI:** 10.1186/s42649-020-00039-2

**Published:** 2020-09-17

**Authors:** Hong-Kyu Kim, Sung-Hoon Kim, Jae-Pyoung Ahn

**Affiliations:** 1 Advanced Analysis Center, Korea Institute of Science and Technology, Seoul, 136-791 Republic of Korea; 2 Mechanical R&D Group, Samsung Electronics, Gyeonggi-do, 16677 Republic of Korea

## Abstract

Deformation twinning, one of the major deformation modes in a crystalline material, has typically been analyzed using generalized planar fault energy **(**GPFE) curves. Despite the significance of these curves in understanding the twin nucleation and its effect on the mechanical properties of crystals, their experimental validity is lacking. In this comparative study based on the first-principles calculation, molecular dynamics simulation, and quantitative in-situ tensile testing of Al nanowires inside a transmission electron microscopy system, we present both a theoretical and an experimental approach that enable the measurement of a part of the twin formation energy of the perfect Al crystal. The proposed experimental method is also regarded as an indirect but quantitative means for validating the GPFE theory.

## Introduction

A twin is a planar defect, which is formed when partial slip occurs under the action of shear stress on more than three consecutive layers of the {111} plane. This defect generation process, also termed as deformation twinning (DT), has received increasing scientific attention due to its effectiveness in blocking the motion of dislocations and thus improving the toughness of metallic materials. The mechanism for the formation of DT has typically been explained by the generalized planar fault energy (GPFE) curve, which depicts the energy landscape corresponding to twin nucleation and subsequent migration of the neighboring planes (Christian and Vítek 1970; Tadmor and Hai 2003; Vitek 1968). Since the GPFE curve quantifies the energy barriers for various fault structures, it has attracted a considerable attention in the modeling of twin formation (Ezaz et al. 2011; Frøseth et al. 2005; Jin et al. 2011; Kibey et al. 2007, 2009; Ogata et al. 2005; Swygenhoven et al. 2004; Wen and Sun 2013; Wu et al. 2006) and in designing microelectromechanical systems (MEMS), which employ nano-scale metals as fundamental structural motifs. The generation of DT is governed by the magnitude of its formation energy, which can be evaluated from the GPFE curve. In contrast to a rich literature on the formation energy of dislocations (Anderson et al. 2017; Hurtado and Ortiz 2012; Jeong et al. 2008; Langer et al. 2010; People 1986; Wang et al. 2016), calculation of the twin formation energy has received only passing mention and the calculation method is not yet fully established. The competition of DS and DT is determined by the relation between the formation energies of the perfect dislocations and twins, and consequently they result in the different deformation behavior activated in materials. Therefore, the calculation and measurement of the twin formation energy is important to understand and expect the deformation behavior of materials.

Apart from its theoretical evaluation using the GPFE curve, the twin formation energy can also be evaluated experimentally using tensile tests. This approach, however, has never been attempted before mainly because of the inherent difficulties encountered with the sample preparation and experimental complexity. First, the samples should be defect-free as the GPFE curve depicts the consecutive activation of the leading (or twinning) partial dislocations i.e., twin formation in a perfect crystal. Furthermore, the structural parameters of the samples such as the crystallographic orientation, stacking fault energy (SFE), Schmid factor, and crystal size, etc., should be within a suitable range so that the combination of these parameters promotes twin formation. Well-grown metal nanowires (NWs), in addition to their extreme crystallinity fulfill the aforementioned stringent requirements and thus are ideal for the experimental measurement of the formation energy of DT. Among various metal NWs, Al NWs due to their high SFE (~ 150 mJ m^− 2^) are known to deform via dislocation slip, but not via DT (Nabarro and Duesbery 2002). In addition, they can be grown in a small size (80–600 nm) with a specific crystallographic orientation and thus, provide an excellent test bed for validating the GPFE theory.

The second problem in the experimental validation of the GPFE theory stems from the complexity and delicacy of the experiment itself. Considering that the GPFE curve depicts the continual change in the fault energy with the formation of twinning partials on the successive layers, it is not easy to measure this subtle change in the energy associated with the movement of the partial dislocations. One way to experimentally measure this energy is using the tensile testing approach as the measured stress-strain curve necessarily contains the energetics corresponding to the defect generation. Based on this argument and considering the small dimensions of the NWs, the in-situ tensile testing of Al NWs inside a transmission electron microscopy (TEM) system is an attractive method for resolving the dissipated energy associated with DT from the measured mechanical response, while simultaneously capturing the instant of the twin formation. The dissipated energy associated with DT measured from the in-situ TEM tensile tests can then be converted to the twin formation energy. The experimentally evaluated value is then compared with the theoretical value obtained from the GPFE curve, thus establishing an indirect but a quantitative approach for validating the GPFE theory.

In the present study, we performed comparative studies to evaluate the twin formation energy using the atomic-scale simulations and the state-of-the-art in-situ TEM tensile test of Al NWs. This was achieved by first deriving an equation that enables the estimation of the twin formation energy from the GPFE curve obtained for a perfect Al crystal using the first-principles calculations.

## Materials and methods

**Fig. 1:**
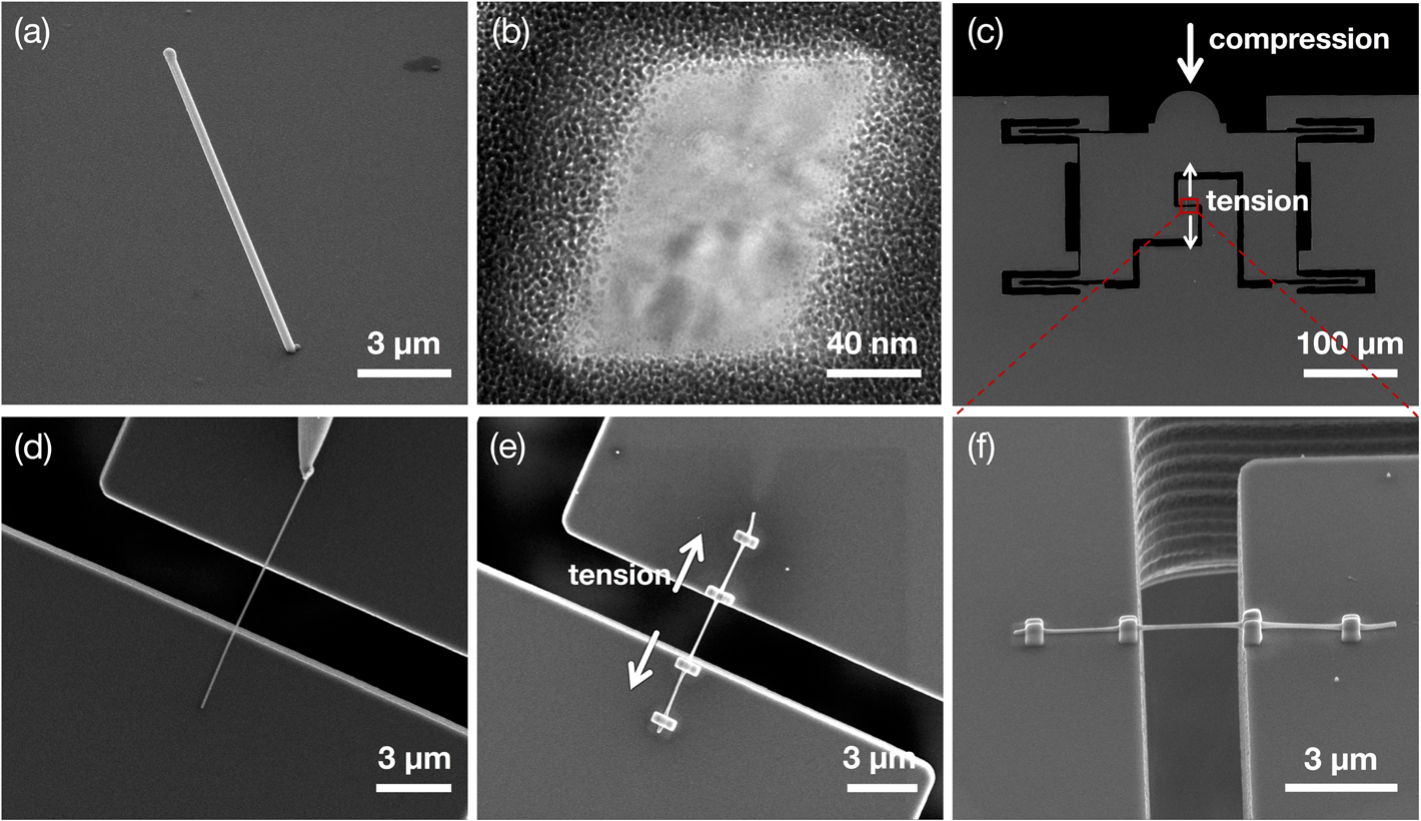
Micrographs of (**a**) Al NW grown on the SiO_2_ substrate and (**b**) its cross-sectional view. **c** Secondary electron image showing the push-to-pull (PTP) loading device, which converts a compressive load to a tensile load. **d**-**f** Magnified views of the rectangle denoted in (**c**); **d** The Al NW transported to the PTP loading device by picking up with the W tip of the nanomanipulator. The NW welded to the PTP device using e-beam assisted Pt deposition viewed from the (**e**) top and (**f**) side

The Al NWs were grown on the SiO_2_ substrate by the stress-induced method reported elsewhere (Lee et al. 2010), as shown in Fig. 1(a). NWs used for in-situ TEM tensile tests were oriented along < 110> and had the diameter of 100–200 nm, the length of 5–20 μm, and a rhombic cross-section with four {111} side facets with an acute angle of 70°, as shown in Fig. 1(b). TEM investigations confirmed that these NWs are single crystalline and nearly defect-free but covered with a thin oxide layer with a thickness of ~ 5 nm. When the NW is welded to the push-to-pull (PTP) loading device, as shown in Fig. 1(c), this device converts a compressive force to a tensile force, enabling the tensile test of NWs in TEM. Before mounting the Al NW to the PTP device, one end of the Al NW sample was first attached using e-beam assisted Pt deposition to a W tip of the nanomanipulator (MM3A, Kleindeck) installed in a FIB system (Quanta 3D, FEI), as shown in Fig. 1(d). This NW was again welded to the upper and lower jigs of the PTP device using e-beam assisted Pt deposition (Figs. 1(e) and (f)). At this stage, a special care is necessary so that its long axis aligns to the tensile direction. The PTP device was then mounted to a picoindenter (PI-95 TEM, Hysitron) capable of measuring the load and displacement. Tensile test was conducted on the Al NW samples at an initial strain rate of 1.2 × 10^− 3^ s^− 1^ at ambient temperature, while the load-displacement data was recorded by simultaneously observing real-time images of the microstructure evolution.

The calculations of the GPFE curve were performed based on the density functional theory (DFT) implemented in the Vienna *Ab-inito* Simulation Package (VASP) (Kresse and Furthmüller 1996; Kresse and Hafner 1993) with projector augmented wave (Blöchl 1994) pseudopotential. For GPFE calculations, the supercell of the Al perfect crystal was constructed of ten layers including two vacuum layers. The orientations of the supercell were parallel to the $\left[2\overline{11}\right],\left[0\overline{1}1\right],$ and [111] directions, corresponding to x-, y-, and z-axes. Upon the sliding of the upper-half layers of the supercell along the direction of the Burgers vector of the partial dislocation, various faults were generated by following the structural transition sequence beginning with the intrinsic stacking fault, extrinsic stacking fault, and twin. During the generation of these faults the associated energy landscape was obtained by generating the GPFE curve.

Next, we performed the tensile testing of the Al NW computationally generated by conventional molecular dynamics (MD) simulations, in order to test its feasibility as an appropriate approach for the evaluation of the twin formation energy and thus validating the GPFE theory. The potential generated from the embedded atom method (Daw and Baskes 1983), as implemented in the LAMMPS (Plimpton 1995) code, was employed to model a computationally generated Al NW with the < 110> longitudinal orientation, rhombic {110} cross-section, and four {111}-side surfaces. The tensile test of the computationally generated Al NW was performed by maintaining a uniform strain loading condition to avoid shock wave loading effects; this was achieved by applying velocity to the individual atoms along the loading direction by varying it linearly from zero at the fixed end to a maximum value at the free end (Park and Zimmerman 2005). The dissipated energy associated with DT measured from the tensile test using MD simulations was compared with that evaluated from the GPFE curve. Inspired by the computational results of the MD simulations, real-world tensile tests were performed on the laboratory-grown < 110> Al NWs using quantitative in-situ TEM to simultaneously record the stress-strain response and the structural evolution sequence associated with DT. The twin formation energy obtained from the stress-strain curve recorded during the in-situ tensile test of the laboratory-grown Al NWs was compared with that evaluated theoretically from the GPFE curve.

## Results and discussion

**Fig. 2:**
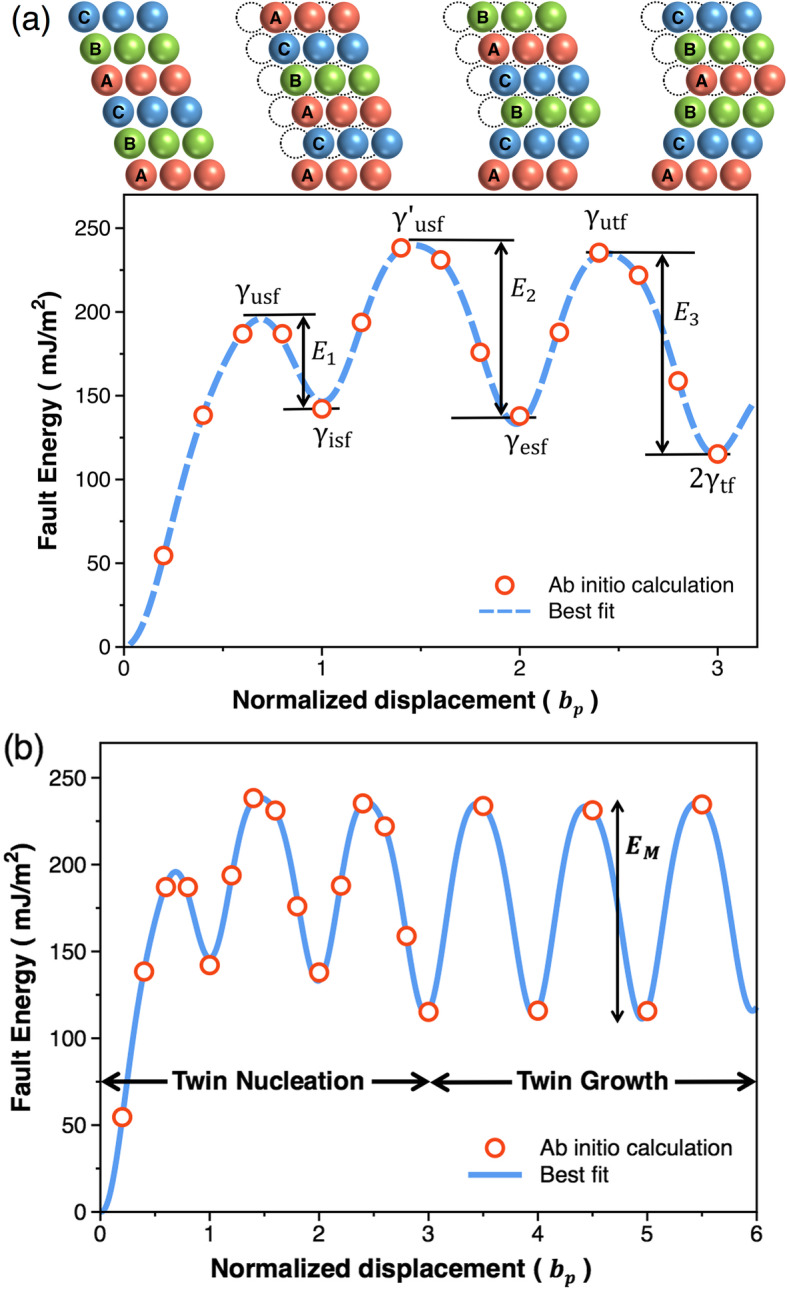
**a** The GPFE curve of a perfect Al crystal calculated using DFT showing the changes in the fault energy associated with the nucleation an embryonic twin consisting of three fault layers. **b** The GPFE curve depicting the growth of the nucleated twin by the consecutive activation of the partial dislocation in the neighboring (111) plane. Here, *b*_*P*_ (= ${a}_0/\sqrt{6}$) is the Burgers vector for the partial dislocation

Figure 2(a) shows the GPFE curve of an Al crystal calculated using the DFT-based first-principles calculation and depicts the continual changes in the fault energy (*γ*) associated with the consecutive activation of twinning partials in the three adjacent (111) planes (i.e., A, B, and C layers). This curve provides a comprehensive description of the nucleation sequence of a twin in terms of energy, while offering a complete picture of the associated structural states. The energy states at the peaks of the GPFE curve correspond to the unstable fault structures, i.e., the unstable stacking fault (*usf*) and unstable twinning fault (*utf*), which are characterized by their energy barriers denoted by *γ*_*usf*_, ${\gamma}_{usf}^{\prime }$, and *γ*_*utf*_, respectively, in Fig. 2(a). With further deformation, each unstable structural state is transformed to a more stable (or metastable) structure, i.e., the intrinsic stacking fault (*isf*), extrinsic stacking fault (*esf*), and twinning fault (*tf*) as characterized by their energy states denoted by *γ*_*isf*_, *γ*_*esf*_, and *γ*_*tf*_, respectively. Despite the difference in the potentials employed and the calculation procedures, the values of various fault energies calculated in this study are very similar to those reported by the previous studies (Brandl et al. 2007; Jin et al. 2011; Lu et al. 2000; Ogata et al. 2005; Sun and Kaxiras 1997) (Table 1), thus confirming the reliability of the present results.

**Table 1:** Various stacking fault energies calculated for the Al crystal. The present results are also compared with those published in the earlier studies. All values are given in mJ m^− 2^

System	*γ*_*usf*_	*γ*_*isf*_(=*γ*_*esf*_)	${\gamma}_{usf}^{\prime }$	*γ*_*utf*_	2*γ*_*tf*_
**Present work**	187	142	238	235	121
Ogata et al. 2005	175	158	235	230	120
Jin et al. 2011	140	112	196	-	100
Brandl et al. 2007	178	146	-	-	-
Lu et al. 2000	224	164	-	-	-
Sun and Kaxiras 1997	224	165	-	-	-

Validation of the GPFE curve requires the measurement of various fault energies listed in Table 1, whose experimental measurements are nearly infeasible. However, the relative differences in the energy levels (indicated as *E*_1_, *E*_2_, and *E*_3_ in Fig. 2) between the unstable and metastable states are measurable quantities because they are the energies dissipated by the crystal and are associated with the nucleation and subsequent migration of a twin (Ogata et al. 2005). Of the two stages of DT, the nucleation stage is accompanied by a series of structural changes, i.e., a transition from the intrinsic stacking fault to the extrinsic stacking fault and finally to the twinning fault. Each structural change is characterized by a transition from an unstable state to a metastable state and requires the associated energies (indicated as *E*_1_, *E*_2_, and *E*_3_ in Fig. 2). The summation of these energies corresponds to the energy (*E*_*N*_) required to nucleate an embryonic twin consisting of three layers of the stacking fault as follows.

1 $$ {\displaystyle \begin{array}{c}{E}_N={E}_1+{E}_2+{E}_3\\ {}=\left({\gamma}_{usf}-{\gamma}_{isf}\right)+\left({\gamma}_{usf}^{\prime }-{\gamma}_{esf}\right)+\left({\gamma}_{utf}-2{\gamma}_{tf}\right)\end{array}} $$

Substitution of the relevant fault energies from Table 1 in Eq. (1) results in *E*_*N*_ = 255 mJ m^− 2^, which corresponds to the energy dissipated during the nucleation of the embryonic twin.

Further application of shear stress causes the nucleated twin to grow (or thicken). This process is achieved by the migration of additional fault layers on the neighboring planes. From the GPFE curve, the twin migration energy (*E*_*M*_) is evaluated as:

2 $$ {E}_M={E}_3=\left({\gamma}_{utf}-2{\gamma}_{tf}\right) $$

In the case of the perfect Al crystal, the magnitude of *E*_*M*_ is 114 mJ m^− 2^ (also denoted by the vertical arrow in Fig. 2(b)). When the consecutive activation of twinning partials occurs on a total of *n*–number of adjacent (111) layers, the nucleated twin grows to a twin consisting of *n*–layers of the stacking fault. In this case, the total energy consumed for the formation of the twin with *n*–layers of the stacking fault (subsequently referenced by ‘*E*(*n*)’) can be calculated as:

3 $$ E(n)={E}_N+\left(n-3\right){E}_M. $$

Equation (3) states that the value of *E*(*n*) is dependent on the inherent properties of the crystal itself (i.e., *E*_*N*_ and *E*_*M*_) and the thickness of the twin as estimated from the number of fault layers (*n*). For a given material, the value of *E*(*n*) depends linearly on the number of fault layers comprising the twin and thus can be evaluated theoretically by substituting the *n*-value in Eq. (3).

The value of *E*(*n*) can also be measured experimentally by resolving the energy from the stress-strain curve recorded from the tensile test; when a stress greater than the yield strength is applied to a material, defects are generated to sustain the deformation at an applied strain rate. This deformation process is accompanied by energy consumption, which is typically revealed by the stress-strain curve obtained by loading and unloading the sample (Nabarro and Duesbery 2002) during the tensile testing. While this technique has traditionally been employed to determine the nucleation energy of dislocations (Cross et al. 2006; Li et al. 2010), the same can also be used to evaluate the value of *E*(*n*). In addition, if the magnitude of the energy evaluated from the tensile test is comparable or similar to that evaluated using Eq. (3), the GPFE theory is validated. Prior to the real-world tensile test using the laboratory-grown Al NWs, the tensile test was performed computationally on an Al NW constructed using MD simulations to test whether the tensile testing is a feasible/suitable approach for validating the GPFE theory.

**Fig. 3:**
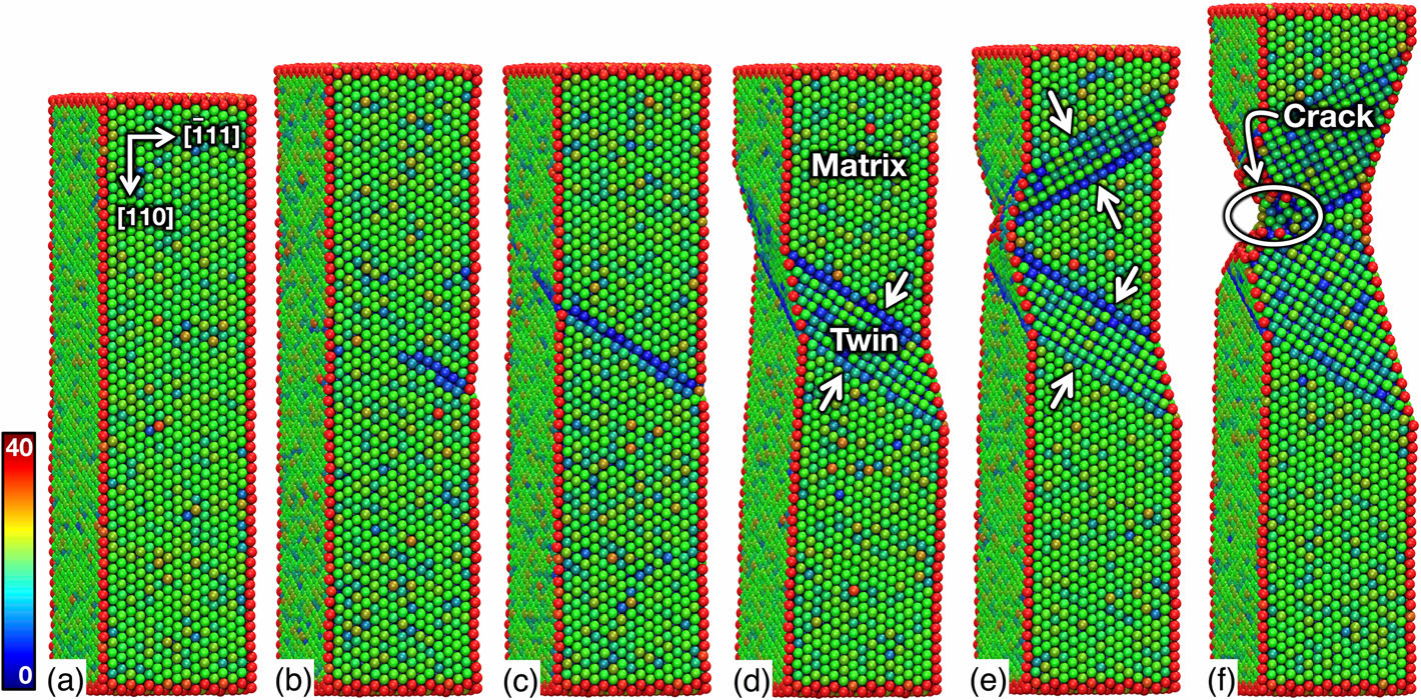
Configuration of the computationally generated Al NW subjected to a tensile strain (휀) of (**a**) 0.02, **b**-**d** 0.05, **e** 0.07, and **f** 0.15, showing a sequence of the twin nucleation and subsequent migration. The atoms are colored to represent the defect structures in the NW according to the values of their centrosymmetry parameter (Kelchner et al. 1998)

Figure 3 shows snapshots of the computationally generated < 110> Al NW captured at various strain stages of deformation. When subjected to a tensile load, the Al NW underwent elastic stretching at the initial stage (Fig. 3(a)) and subsequently suffered from yield at ~ 3.2 GPa (or ε ≈ 5%) with the formation of a stacking fault (Fig. 3(b)). This stacking fault is initially nucleated from the acute-angle corner of the rhombic-shaped {111} plane and propagates along the < 211> direction (Fig. 3(c)). With further deformation, additional fault layers are formed on the successive planes leading to the formation of a twin as shown in Fig. 3(d). Interestingly, once the first twin is formed, it does not grow beyond a certain thickness. Rather, a second twin is formed in the regions in the vicinity of the first twin (Fig. 3(e)), which upon further deformation overlap at an angle of 70.5°, self-lock each other, and cause pronounced strain localization (Fig. 3(f)).

**Fig. 4:**
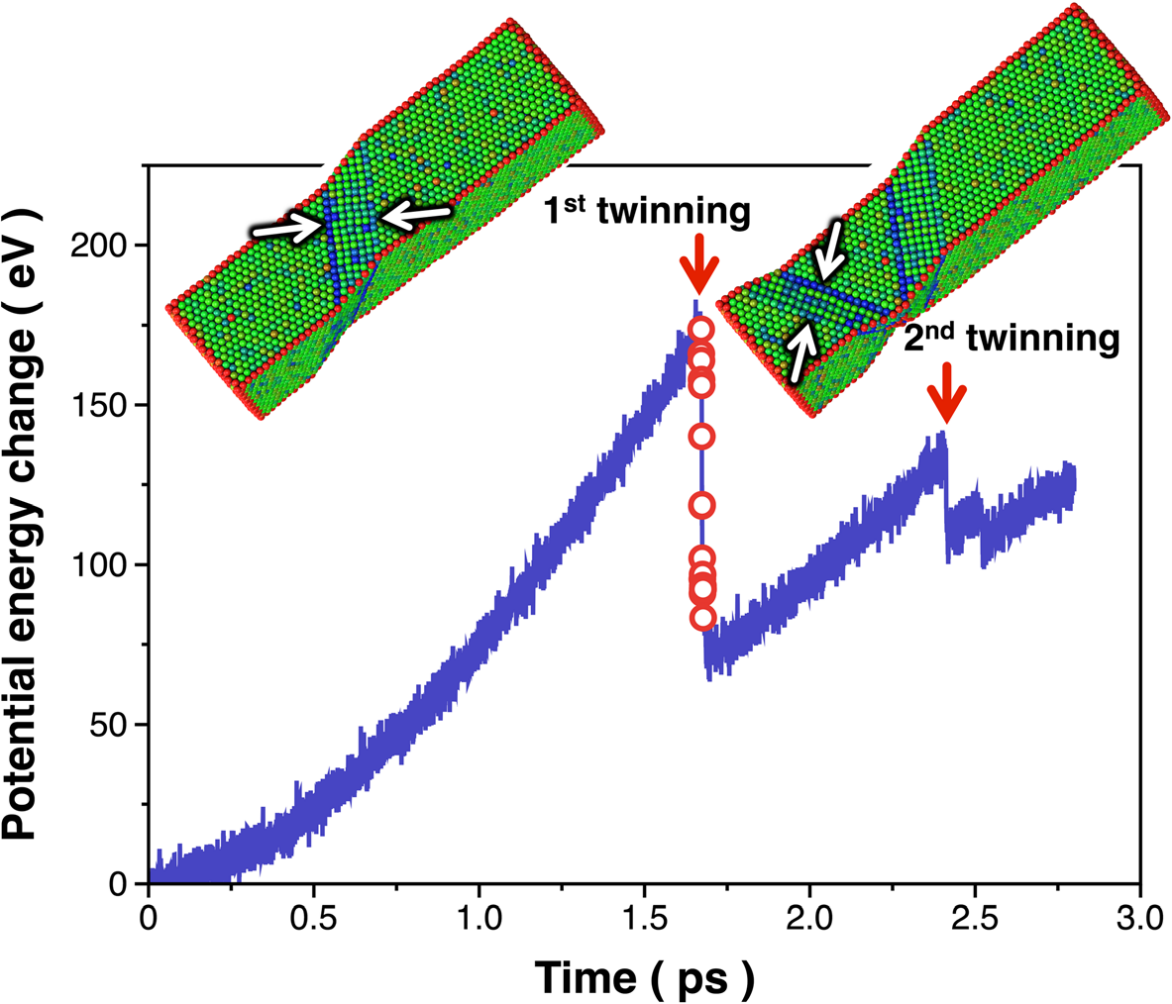
Changes in the potential energy associated with the formation of twins in the computationally generated Al NW. The potential energy of the Al NW was calculated as a function of the time step, showing a significant drop in the potential energy at times when the twinning events occur

The structural evolution of the Al NW (Fig. 3) is driven by the externally applied force, which can be evaluated in terms of the changes in the potential energy of the NW. Figure 4 shows the changes in the potential energy of the computationally generated Al NW calculated as a function of the time step for deformation using MD simulations. The potential energy of the Al NW increases with increasing time and drops abruptly when the twinning events occur (as denoted by the arrows in Fig. 4); for example, the formation of the first twin consisting of seven fault layers (Fig. 3(d)) is accompanied by a reduction of ~ 88 eV (at the time step of 1.7 ps in Fig. 4) in the potential energy. In other words, this is the energy dissipated by the NW as a result of the formation of the first twin with *n* = 7. This change in the potential energy can be converted to the twin formation energy, i.e., *E*(7) by normalizing it with the area (~ 17.7 nm^2^) of the twin boundary plane, leading to a twin formation energy of ~ 691 mJ m^− 2^. As discussed earlier, given the number of fault layers comprising the twin, the value of *E*(*n*) can also be evaluated from Eq. (3) based on the GPFE theory. The value of *E*(7) (= *E*_*N*_ + (7 − 3)*E*_*M*_) is evaluated to be 711 mJ m^− 2^, which is very close to that (691 mJ m^− 2^) determined from the tensile test of the computationally generated Al NW. This quantitative analysis provides a valuable insight as to why the in-situ TEM tensile testing of the Al NW can be a plausible means for evaluating the twin formation energy thus validating the GPFE theory. Inspired by the result of the tensile-test of the computationally generated NW, we carried out a real-world tensile testing of the laboratory-grown Al NW using quantitative in-situ TEM to elucidate whether this technique can also produce a self-consistent result.

**Fig. 5:**
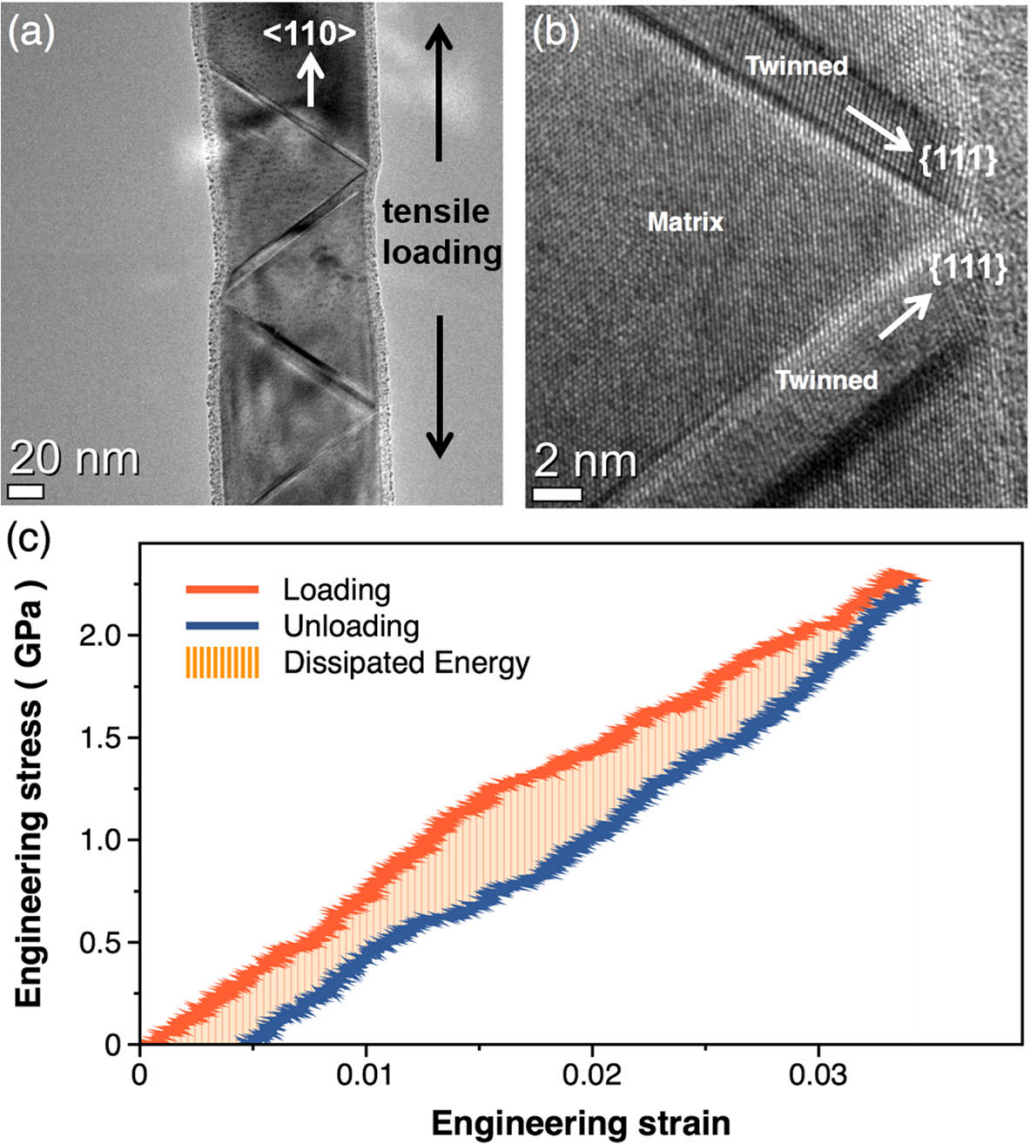
**a** Representative TEM image of the tensile-test < 110> Al NW, showing the formation of four twins with a zig-zag configuration. **b** Magnified view of the twinned region showing the interlocking configuration of the twins. **c** Stress-strain curve of the Al NW recorded by unloading the sample. Note that the Al NW was observed to yield with the formation of twins at *σ*= 2.3 GPa (equivalently, at *ε* = 3.4%)

Figure 5(a) is the TEM micrograph of the laboratory-grown < 110> Al NW subjected to a deformation to ε = 3.4% showing the presence of four twins as shown in Fig. 1. This result provides the first direct and unambiguous evidence that DT indeed occurs in a perfect < 110> Al crystal thus confirming the simulation results from a previous study (Jo et al. 2014). However, it should be noted that as compared to the twin migration behavior observed in most fcc-metal NWs with low SFEs (Hwang et al. 2015; Lee et al. 2014; Seo et al. 2011, 2013), the twins formed in the Al NW did not propagate through the entire volume of the NW. Instead, they grew to a limited thickness by forming a zig-zag configuration in a manner similar to that observed by the MD simulations (see Fig. 3). When observed at high magnification (Fig. 5(b)), these twins formed the Σ3 {111} twin boundary, which is inclined at an angle of 55^o^ with respect to the tensile direction. Upon further deformation, this zig-zag configuration formed between twins limits the growth and the propagation of each twin and forces the twins to be self-locked each other. This limited configuration caused a pronounced strain localization in an area interconnected between two twins, thus making it infeasible for the NW to carry a plastic strain in a measurable quantity.

As soon as the twins formed, the tensile test was interrupted by unloading the sample to obtain the stress-strain curve as shown in Fig. 5(c). The area enclosed by the stress-strain curve in Fig. 5(c) corresponds to the change in the internal energy per unit volume of the laboratory-grown Al NW (~ 31.2 meV nm^− 3^). When the partial dislocation is nucleated, this dislocation moves along the {111} plane and simultaneously the stacking fault is propagated. In this propagation process, the energy required for the propagation is linearly related to the area of the {111} plane. Thus, if the measured formation energy is divided by the area of related planes, it is no longer necessary to consider the size of the NWs. Furthermore, the fault energies and their combinations (*E*_*N*_*, E*_*M*_*, E(n)*) are in units of joule per square metre (mJ m^− 2^), and this is equivalent to the units of the volumetric energy rescaled by the area. Consequently, this rescaled volumetric energy can be compared to the measured toughness directly. In this perspective, this volumetric energy is rescaled to the twin formation energy by multiplying it with the volume (~ 4.2 × 10^7^ nm^3^) of the NW followed by a division by the area (~ 1.8 × 10^4^ nm^2^) of the twin boundary planes; this results in a twin formation energy of ~ 11,240 mJ m^− 2^, which is the total energy responsible for the formation of the four twins shown in Fig. 5(a). Since this energy can be regarded as the energy dissipated during the formation of the twins, we next compared this experimentally measured energy with that calculated using Eq. (3). Given the thickness (4.1, 5.1, 7.4, and 9.0 nm) of the four twins, the approximate number of fault layers comprising each twin can be estimated (*n* = 14, 18, 25, and 31, respectively). Therefore, using these experimental values of *n* and the values of *E*_*N*_ and *E*_*M*_ obtained from the GPFE curve the total energy dissipated due to the formation of the four twins (Σ*E*(*n*)) is calculated to be 9684 mJ m^− 2^. Upon comparison of the two values of the twin formation energy, the experimentally measured value is found to be larger than the theoretical value by 16%. One possible reason for this mismatch probably arises from the interaction between the neighboring twins during the tensile test; when a new twin forms from the free surface of the NW near the pre-formed twin, the newly formed twin thickens until its migrating fault layer meets that of the pre-formed twin. This interaction between the twins may cause an additional stress acting on the Al NW during the tensile testing, which was not taken into consideration while predicting the yield strength using the GPFE curve. Another possible reason is maybe related to changes of the density of dislocations. During the in-situ TEM tensile testing, the density of dislocations increases gradually, and this will affect the toughness of the Al NWs resulting in the increase of the energy measured experimentally. Despite the difference in the magnitude, the value of *E*(*n*) predicted using the GPFE curve exhibits, in general, a close correspondence to that measured experimentally by the in-situ TEM tensile test.

## Conclusions

In summary, both theoretical and experimental methods that enable the assessment of the energy required for the formation of twins in the Al NWs were demonstrated. By knowing the instant of the twin formation and simultaneously measuring the stress-strain response of the Al NWs during the loading-unloading experiment inside a quantitative in-situ TEM system, we could resolve the dissipated energy, which in turn can be converted to *E*(*n*). This experimentally measured energy was found to be in agreement with that calculated theoretically from the GPFE curve, thus validating the GPFE theory. According to the theoretical and experimental results obtained in this study, it is concluded that the combination of in-situ TEM tensile testing and atomic simulations enables a deeper understanding of twin formation in various fcc-metal NWs. According to our works reported recently (Kim et al. 2018a, b, 2020), however, the formation energy of DT includes not only the fault energy shown in the GPFE curve, but also the nucleation energy required for the formation of dislocations. Thus, when calculating the formation energy of DT, this term of the nucleation energy has to be taken account for the precise measurement of the formation energy of DT, but we leave this as a future work.

## Data Availability

The datasets used and/or analyzed during the current study are available from the corresponding author on reasonable request.

## Abbreviations

DT: Deformation twinning
GPFE: Generalized Planar Fault Energy
MD: Molecular Dynamics
NW: Nanowire
TEM: Transmission Electron Microscopy
MEMS: Microelectromechanical Systems
SFE: Stacking Fault Energy
VASP: Vienna Ab-initio Simulation Package

## References

[CR1] Anderson PM, Hirth JP, Lothe J (2017). Theory of dislocations.

[CR2] Blöchl PE (1994). Projector augmented-wave method. Phys. Rev. B.

[CR3] Brandl C, Derlet P, Swygenhoven HV (2007). General-stacking-fault energies in highly strained metallic environments: Ab initio calculations. Phys. Rev. B.

[CR4] Christian JW, Vítek V (1970). Dislocations and stacking faults. Rep. Prog. Phys..

[CR5] Cross GLW, Schirmeisen A, Grütter P, Dürig UT (2006). Plasticity, healing and shakedown in sharp-asperity nanoindentation. Nat. Mater..

[CR6] Daw MS, Baskes MI (1983). Semiempirical, quantum mechanical calculation of hydrogen Embrittlement in metals. Phys. Rev. Lett..

[CR7] Ezaz T, Sehitoglu H, Maier HJ (2011). Energetics of twinning in martensitic NiTi. Acta Mater..

[CR8] Frøseth AG, Derlet PM, Swygenhoven HV (2005). Twinning in nanocrystalline fee metals. Adv. Eng. Mater..

[CR9] Hurtado DE, Ortiz M (2012). Surface effects and the size-dependent hardening and strengthening of nickel micropillars. J. Mech. Phys. Solids.

[CR10] Hwang B, Kang M, Lee S, Weinberger CR, Loya P, Lou J, Oh SH, Kim B, Han SM (2015). Effect of surface energy on size-dependent deformation twinning of defect-free Au nanowires. Nanoscale.

[CR11] Jeong BW, Ihm J, Lee G-D (2008). Stability of dislocation defect with two pentagon-heptagon pairs in graphene. Phys. Rev. B.

[CR12] Jin ZH, Dunham ST, Gleiter H, Hahn H, Gumbsch P (2011). A universal scaling of planar fault energy barriers in face-centered cubic metals. Scr. Mater..

[CR13] Jo M, Koo YM, Lee B-J, Johansson B, Vitos L, Kwon SK (2014). Theory for plasticity of face-centered cubic metals. Proc. Natl. Acad. Sci..

[CR14] Kelchner CL, Plimpton SJ, Hamilton JC (1998). Dislocation nucleation and defect structure during surface indentation. Phys. Rev. B.

[CR15] Kibey S, Liu JB, Johnson DD, Sehitoglu H (2007). Predicting twinning stress in fcc metals: Linking twin-energy pathways to twin nucleation. Acta Mater..

[CR16] Kibey S, Wang L, Liu J, Johnson H, Sehitoglu H, Johnson D (2009). Quantitative prediction of twinning stress in fcc alloys: application to Cu-Al. Phys. Rev. B.

[CR17] Kim H-K, Kim S-H, Ahn J-P, Lee J-C (2018). Deformation criterion for face-centered-cubic metal nanowires. Mater. Sci. Eng..

[CR18] Kim S-H, Kim H-K, Seo J-H, Whang D-M, Ahn J-P, Lee J-C (2018). Deformation twinning of ultrahigh strength aluminum nanowire. Acta Mater..

[CR19] Kim S-H, Park J-H, Kim H-K, Ahn J-P, Whang D-M, Lee J-C (2020). Twin boundary sliding in single crystalline Cu and Al nanowires. Acta Mater..

[CR20] Kresse G, Furthmüller J (1996). Efficiency of ab-initio total energy calculations for metals and semiconductors using a plane-wave basis set. Comput. Mater. Sci..

[CR21] Kresse G, Hafner J (1993). Ab initio molecular dynamics for open-shell transition metals. Phys. Rev. B.

[CR22] Langer JS, Bouchbinder E, Lookman T (2010). Thermodynamic theory of dislocation-mediated plasticity. Acta Mater..

[CR23] Lee JW, Kang MG, Kim BS, Hong BH, Whang D, Hwang SW (2010). Single crystalline aluminum nanowires with ideal resistivity. Scr. Mater..

[CR24] Lee S, Im J, Yoo Y, Bitzek E, Kiener D, Richter G, Kim B, Oh SH (2014). Reversible cyclic deformation mechanism of gold nanowires by twinning-detwinning transition evidenced from in situ TEM. Nat. Commun..

[CR25] Li S, Ding X, Li J, Ren X, Sun J, Ma E (2010). High-efficiency mechanical energy storage and retrieval using interfaces in nanowires. Nano Lett..

[CR26] Lu G, Kioussis N, Bulatov VV, Kaxiras E (2000). Generalized-stacking-fault energy surface and dislocation properties of aluminum. Phys. Rev. B.

[CR27] Nabarro FR, Duesbery MS (2002). Dislocations in solids.

[CR28] Ogata S, Li J, Yip S (2005). Energy landscape of deformation twinning in bcc and fcc metals. Phys. Rev. B.

[CR29] Park H, Zimmerman J (2005). Modeling inelasticity and failure in gold nanowires. Phys. Rev. B.

[CR30] People R (1986). Physics and applications of GexSi1-x/Si strained-layer heterostructures. IEEE J. Quantum Electron..

[CR31] Plimpton S (1995). Fast parallel algorithms for short-range molecular dynamics. J. Comput. Phys..

[CR32] Seo J-H, Park HS, Yoo Y, Seong T-Y, Li J, Ahn J-P, Kim B, Choi I-S (2013). Origin of size dependency in coherent-twin-propagation-mediated tensile deformation of noble metal nanowires. Nano Lett..

[CR33] Seo JH, Yoo Y, Park NY, Yoon SW, Lee H, Han S, Lee SW, Seong TY, Lee SC, Lee KB, Cha PR, Park HS, Kim B, Ahn JP (2011). Superplastic deformation of defect-free au nanowires via coherent twin propagation. Nano Lett..

[CR34] Sun Y, Kaxiras E (1997). Slip energy barriers in aluminium and implications for ductile-brittle behaviour. Philos. Mag..

[CR35] Swygenhoven HV, Derlet PM, Frøseth AG (2004). Stacking fault energies and slip in nanocrystalline metals. Nat. Mater..

[CR36] Tadmor EB, Hai S (2003). A Peierls criterion for the onset of deformation twinning at a crack tip. J. Mech. Phys. Solids.

[CR37] Vitek V (1968). Intrinsic stacking faults in body-centered cubic. Acta Metallurgica Sin. Engl. Lett..

[CR38] Wang L, Liu Z, Zhuang Z (2016). Developing micro-scale crystal plasticity model based on phase field theory for modeling dislocations in heteroepitaxial structures. Int. J. Plast..

[CR39] Wen YF, Sun J (2013). Generalized planar fault energies and mechanical twinning in gamma TiAl alloys. Scr. Mater..

[CR40] Wu X, Zhu YT, Ma E (2006). Predictions for partial-dislocation-mediated processes in nanocrystalline Ni by generalized planar fault energy curves: an experimental evaluation. Appl. Phys. Lett..

